# Identifying older adults at risk for dementia based on smartphone data obtained during a wayfinding task in the real world

**DOI:** 10.1371/journal.pdig.0000613

**Published:** 2024-10-03

**Authors:** Jonas Marquardt, Priyanka Mohan, Myra Spiliopoulou, Wenzel Glanz, Michaela Butryn, Esther Kuehn, Stefanie Schreiber, Anne Maass, Nadine Diersch

**Affiliations:** 1 Multimodal Neuroimaging Group, German Center for Neurodegenerative Diseases (DZNE), Magdeburg, Germany; 2 Faculty of Computer Science, Otto-von-Guericke-University Magdeburg, Magdeburg, Germany; 3 German Center for Neurodegenerative Diseases (DZNE), Magdeburg, Germany; 4 Hertie Institute for Clinical Brain Research (HIH), Tübingen, Germany; 5 Institute of Cognitive Neurology and Dementia Research (IKND), Otto-von-Guericke University, Magdeburg, Germany; 6 Translational Imaging of Cortical Microstructure, German Center for Neurodegenerative Diseases (DZNE), Tübingen, Germany; 7 Department of Neurology, Otto-von-Guericke University Magdeburg, Magdeburg, Germany; 8 Center for Behavioral Brain Sciences (CBBS), Otto-von-Guericke University Magdeburg, Magdeburg, Germany; 9 Institute of Biology, Otto-von-Guericke-University Magdeburg, Magdeburg, Germany; Research Institute of the McGill University Health Centre, McGill University, CANADA

## Abstract

Alzheimer’s disease (AD), as the most common form of dementia and leading cause for disability and death in old age, represents a major burden to healthcare systems worldwide. For the development of disease-modifying interventions and treatments, the detection of cognitive changes at the earliest disease stages is crucial. Recent advancements in mobile consumer technologies provide new opportunities to collect multi-dimensional data in real-life settings to identify and monitor at-risk individuals. Based on evidence showing that deficits in spatial navigation are a common hallmark of dementia, we assessed whether a memory clinic sample of patients with subjective cognitive decline (SCD) who still scored normally on neuropsychological assessments show differences in smartphone-assisted wayfinding behavior compared with cognitively healthy older and younger adults. Guided by a mobile application, participants had to find locations along a short route on the medical campus of the Magdeburg university. We show that performance measures that were extracted from GPS and user input data distinguish between the groups. In particular, the number of orientation stops was predictive of the SCD status in older participants. Our data suggest that subtle cognitive changes in patients with SCD, whose risk to develop dementia in the future is elevated, can be inferred from smartphone data, collected during a brief wayfinding task in the real world.

## Author summary

Dementia poses a significant challenge for healthcare systems worldwide. For interventions and treatments to be effective, the early detection of cognitive changes associated with the disease will be crucial. Leveraging recent advances in mobile consumer technologies, we investigated the potential of smartphone data to identify at-risk individuals in a real-life scenario. Specifically, we tracked wayfinding behavior in a memory clinic sample of patients with subjective cognitive decline (SCD) who still scored normally in standard neuropsychological assessments, as well as cognitively healthy older and younger adults. Participants were asked to navigate to different locations on the local university campus, guided by a mobile application. From GPS and user input data, we extracted different performance measures to determine differences between the groups. We show that the number of orientation stops can predict SCD status in older participants. Thus, this study introduces a promising approach to detect cognitive decline using digital data obtained during a real-world wayfinding task, with potential implications for early dementia identification and monitoring, ultimately improving the diagnosis of AD and aiding decision-making in primary healthcare.

## Introduction

Currently, about 58 million people around the world are living with dementia, including 32 million cases of Alzheimer’s disease (AD) as the most common form of dementia. Additionally, 69 (315) million are estimated to be in the prodromal (preclinical) stage of the disease [1]. Moreover, the prevalence of dementia is expected to triple by 2050, due to population growth and rising life expectancies in many countries [2]. This constitutes a major burden on societies and healthcare systems by causing enormous direct (e.g., skilled nursing or professional medical care) as well as indirect (e.g., informal caregivers) costs [3,4].

Until now, there are no treatments available to cure the disease [5], although several drugs have been shown to alter the disease trajectory in phase-3 clinical trials. For example, the now FDA-approved anti-amyloid antibody *lecanemab* has been shown to slow down cognitive decline in older adults at early AD stages (mild cognitive impairment, MCI, or mild dementia due to AD) compared with a placebo group [6]. This necessitates the development of novel diagnostic tools that assess cognitive functioning in individuals who are still asymptotic in standard neuropsychological assessments but might already show subtle cognitive changes that are associated with an elevated risk to develop dementia and, ultimately, might be suggestive of the accumulation of amyloid and tau in certain brain areas [7]. Identifying individuals who are at-risk for dementia as early as possible will also help to evaluate which individuals need further diagnostics and also decide on the initiation of disease-modifying interventions to extend the time they can live independently in their community.

In recent years, as mobile and wearable consumer technologies are becoming widely accessible and are also increasingly adopted by older age groups [8], the interest in digital cognitive assessments for a cost-efficient and easy-to-administer diagnosis of neurological diseases is rapidly growing [9–12]. One advantage is the possibility to collect data remotely and thereby increasing the ecological validity of the test results [11,13]. In addition, multi-dimensional data from different sensors of the devices can be recorded at a high frequency, which enables the detection of complex multivariate changes in behavior that evolve with disease progression [14,15]. With respect to the diagnosis of AD, first promising attempts were made to utilize digital tools for the detection of MCI-grade episodic memory impairments, which are linked to computations in sub-regions of the medial temporal lobe (MTL) where tau typically starts to accumulate many years before the clinical stage of AD manifests [16,17].

Another cognitive ability, which is severely compromised in AD patients and mainly linked to neuronal resources in the MTL, is the ability to form a spatial representation of the environment, to determine one’s own location, and to navigate successfully from one place to another (i.e., spatial navigation) [18–22]. Evidence from a growing number of experimental studies, often assessing navigational performance using virtual reality (VR) setups, indicates that spatial navigation tests may show a higher sensitivity and specificity for identifying individuals at-risk for AD than episodic memory tests [18]. For example, Bierbrauer and colleagues [23] reported that the ability to estimate the current position in the environment based on information about previous positions (i.e., path integration), which is linked to computations of spatially-tuned cells (grid cells) in the entorhinal cortex, is compromised in adults who are at genetic risk for AD (APOE e4 carriers, see also Kunz and colleagues [24]). In addition to dementia due to AD, there are also other common types of dementia, including vascular dementia, frontotemporal dementia, and others [25]. Even within AD, different sub-types of the disease have been identified, which are related to distinct trajectories of tau deposition, such as the limbic-predominant or medial temporal lobe-sparing sub-type [26]. Spatial navigation is a multifaceted cognitive process encompassing action planning, path integration, goal-directed behavior, and self-monitoring processes, which rely on different neuronal networks. While the MTL plays a crucial role, other brain regions, particularly parietal and prefrontal areas associated with attention and executive function, are also integral to this complex behavior [18,19,27].

Thus, different dementia causes or AD phenotypes might become evident as impairments in specific cognitive sub-processes of spatial navigation [26,28,29]. In line with this, Chen and colleagues [30] found that individuals, who report subjective cognitive decline (SCD) but do not yet show any impairments in conventional episodic memory tests, are performing worse in a virtual navigation task and show reduced functional connectivity in relevant brain areas, including the retrosplenial cortex, the hippocampus, and the prefrontal cortex, relative to healthy controls.

While not all individuals with SCD will progress to AD or develop dementia, previous studies have linked SCD to an elevated risk to progress to mild cognitive impairment (MCI), dementia, and AD [31–34]. Additionally, SCD has been linked to increased severity of AD pathology in the brain, especially in areas of the MTL [33,35,36], as well as to a higher genetic risk for AD (APOE status) [37]. Furthermore, Tangen and colleagues showed that spatial navigation ability, assessed via the floor maze test, was predictive of progression to dementia at a 2 and 4-year follow up in SCD patients while task performance was also related to MTL, parietal, and prefrontal cortex volume [38]. In addition, AD pathology in SCD patients predicts future declines in memory, global cognition, and executive function [39].

Despite the potential of VR-based tasks to detect AD-related cognitive impairment, the technology requires extensive training and supervision, is cost-intensive, and testing participants in VR settings poses additional challenges such as the discrepancy between visual and body-based cues, which are both important for efficient navigation. Moreover, the susceptibility to motion sickness is increased in older age groups, further limiting the diagnostic applicability of VR [40]. Moreover, most of the previous studies investigating changes in spatial navigation focused on accuracy (e.g., pointing errors) as outcome measure, without assessing the informative value of movement trajectories. First evidence from human and animal studies, however, suggests that health-related information can be inferred from movement trajectories. For example, Coughlan and colleagues [41] showed that APOE e4 carriers travel longer distances in the mobile wayfinding game “SeaHeroQuest” compared with non-carriers (for similar findings in rodent models of AD see Ying and colleagues [42]).

Here, we introduce a novel smartphone-assisted wayfinding task to investigate the diagnostic value of movement trajectories and related information in different participant groups during navigation in the real world. We aimed to identify which patterns in the data indicate subtle changes in cognition in a memory clinic sample of patients with SCD and are therefore potentially related to an increased risk to develop dementia in the future. More precisely, we assessed their navigational abilities in comparison to the performance of healthy younger and older participants during a brief wayfinding task, resembling aspects of a typical everyday behavior, namely, finding places in the immediate surroundings that are displayed on a map, guided by our in-house developed mobile application “Explore”. In this way, we circumvent limitations of VR-based spatial navigation tasks. Moreover, in contrast to existing digital assessments of cognitive impairment, we consider passive data (e.g., GPS data) in combination with active data (e.g., user inputs) as outcome measures (cf. ref. [43]). The potential of passive data has recently been substantiated by Ghosh and colleagues [44] who equipped a group of AD patients and healthy controls with a GPS tracker and recorded their movement trajectories whenever they left their home (accompanied as well as unaccompanied) over a period of two weeks. The authors extracted multiple parameters from the GPS data (e.g., distance from home, entropy, duration of stops, segment similarity and complexity) and showed that some of these parameters differed between the two groups and could be used to predict the participant’s disease status. Whether potential cognitive changes in individuals who report SCD but are still clinically normal can be detected by a smartphone-based wayfinding task, performed in the real world, remains unknown.

Based on findings that navigating to familiar destinations remains relatively unimpaired with advancing age [45,46], we asked our participants to find locations, which were not known to them prior to testing and which were unlikely to be part of their everyday navigation routes, on the relatively confined medical campus area of the Magdeburg university. In line with previous studies, differences between the three groups in our study might become particularly evident for the covered distances during wayfinding [41]. Alternatively, group difference might emerge on measures that capture the participants’ cognitive processing demands and uncertainty about where to go in order to reach a destination, in line with findings showing that impairments in spatial navigation may lead to behaviors aiming at lowering cognitive efforts during task performance (i.e., viewing the map more often during wayfinding or briefly stopping during navigation in order to orient) [47]. Answering these questions will offer critical information on how to use mobile data to detect behavioral patterns that are associated with an elevated risk to develop dementia.

## Results

In this study, findings are reported from 24 younger adults, 25 cognitively healthy older adults, and 23 patients with subjective cognitive decline (SCD). The SCD patients were recruited based on referrals to the DZNE memory clinic (as opposed to recruitment advertisements) and underwent an extensive neuropsychological assessment by a neurologist at the clinic, following the criteria of Jessen and colleagues [31]. The SCD patients performed within age-, sex-, and education-adjusted norms in the consortium to establish a registry for Alzheimer’s disease test battery (CERAD) [48], consisting of several subtests that measure different aspects of cognitive functioning (e.g., verbal fluency, episodic memory, executive functioning).

**Fig 1:**
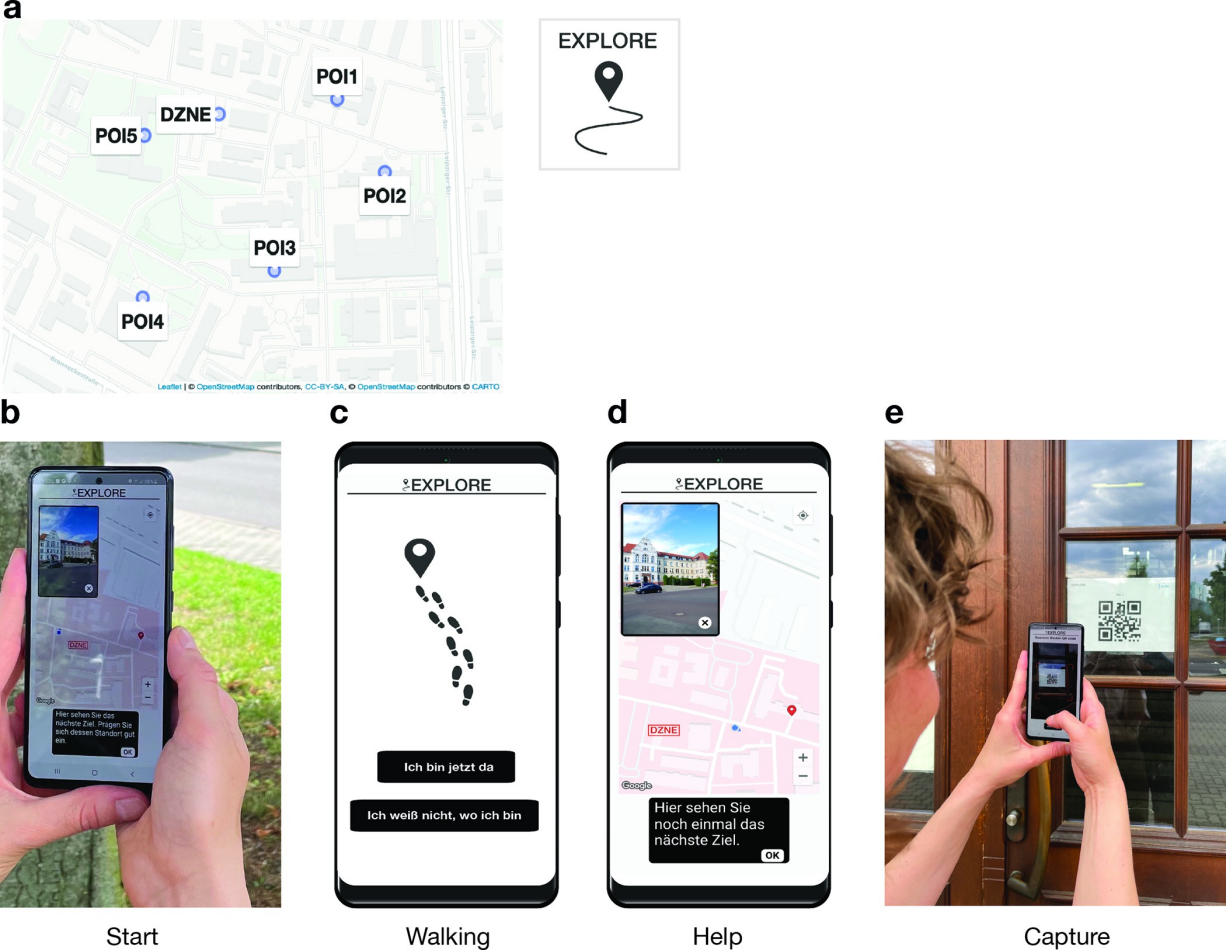
Mobile wayfinding task implemented in the “Explore” app. (a) Starting from the DZNE, participants had to find five points-of-interest (POIs) on the surrounding campus area. Base map data is copyrighted to OpenStreetMap contributors under the Open Database License (https://www.openstreetmap.org/copyright/en). Base map style is copyrighted to Carto (www.carto.com) under a CC-BY 4.0 license (https://github.com/CartoDB/basemap-styles/blob/master/LICENSE.md). (b) For each POI, a map was displayed at the start of the track, showing the current location of the participant and the POI location as well as a picture of the POI (start phase). (c) During walking, the map was not shown (walking phase). (d) If needed, participants could view the map again by calling the help function (help phase). (e) A track was successfully completed when a QR code, which was placed at the entrance door of the POI, was scanned with the phone camera (capture phase). The phone mockup in Fig 1C–1D was designed by Freepik (http://www.freepik.com).

Participants performed a mobile wayfinding task on the campus area around the German Center for Neurodegenerative Diseases (DZNE) in Magdeburg, Germany. Guided by our newly developed smartphone application “Explore”, participants were asked to walk from the DZNE to five salient buildings on the campus (points-of-interest, Fig 1A), while their GPS data were recorded (latitude, longitude, and timestamp). The points-of-interest (POI) had to be found consecutively, and the whole route covered approximately 820 m. At the beginning of each walking track, participants saw a map on the smartphone, showing their own location and the POI location as well as a picture of the POI (Fig 1B). Participants were instructed to close the map before they started walking and to find the POI independently (Fig 1C). Participants had the possibility to view the map again, if needed, and the number of times they called this help function was recorded for each track (Fig 1D). A QR code, which was placed at the entrance door of the target POI, had to be scanned with the phone camera once the participant arrived at the POI. This confirmed the completion of the current track and initiated the corresponding procedure for the next track (Fig 1E). All of the participants were smartphone owners, did not report any mobility impairments and possessed comparable levels of familiarity with the campus area, as assessed in a screening session before testing (Table 1).

**Table 1:** Sample characteristics (descriptives; mean scores ± SD) of the participant groups.

	YA	OA	SCD
N	24	25	23
Age	24.5 ± 2.36	65.7 ± 4.02	66.2 ± 6.54
No of female	12	13	12
Campus familiarity (max. score: 28)	12.7 ± 9.15	14.1 ± 8.77	8.9 ± 6.27
Life-Space Assessment (max. score: 120)	81.0 ± 12.1	84.2 ± 16.0	84.5 ± 14.1
Cognitive screening scores	--	MoCA: 28.5 ± 1.12	MMSE: 28.8 ± 1.31 CERAD: 0.17 ± 0.54 min = -0.82

The CERAD composite score was calculated using the age-, sex-, and education-corrected z-scores from six different sub-tests (Boston Naming Test, verbal fluency, word list learning, word list recall, word list savings, and constructional praxis, see Chandler et al. [49]). The groups (YA: younger adults; OA: healthy older adults; SCD: patients with subjective cognitive decline) did not differ in the listed attributes. Age differences between healthy older adults and SCD patients were tested using a two-sided Welch two-sample t-test, t (35.95) = -0.34, p = .736, d = 0.10. Sex differences were tested using a χ2 test, χ2 (2) = 0.03, p = .986, d = 0.04. Differences in campus familiarity were tested using a Kruskal-Wallis rank-sum test, χ2 (2) = 4.38, p = .112, η^2^ = 0.03; Life-space assessment score differences using an analysis of variance, F(2,69) = 0.42, p = .660, η^2^ = 0.01.

### Differences in GPS trajectories do not distinguish between participant groups

**Fig 2:**
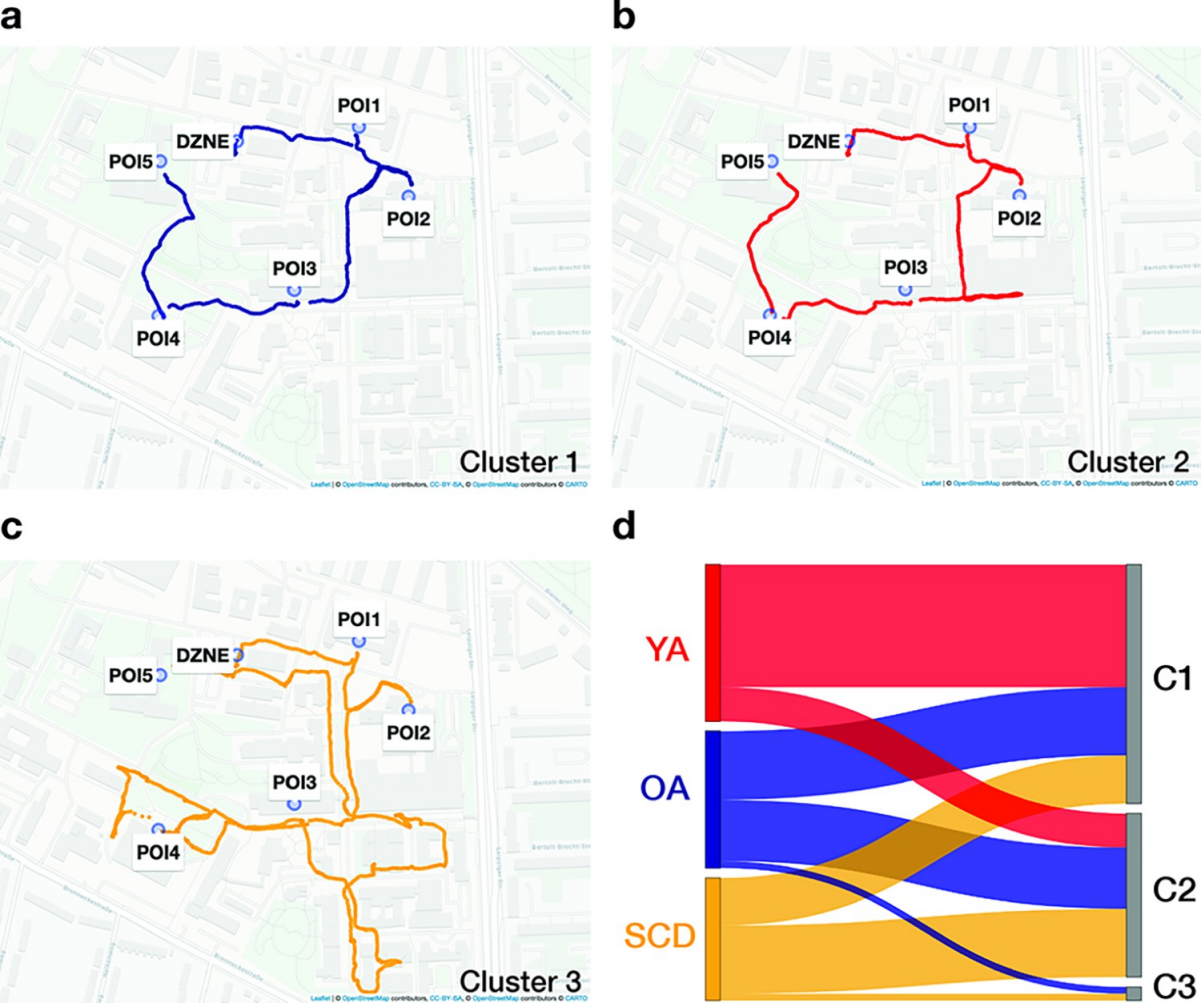
Three wayfinding styles in our sample as identified by a k-medoids clustering analysis using differences in GPS trajectories as input features. GPS trajectory of a representative member (cluster medoid) of the (a) first, (b) second, and (c) third cluster. (d) Sankey diagram showing the distribution of our participant classes in each wayfinding cluster. Red: younger adults; blue: healthy older adults; yellow: patients with subjective cognitive decline. Base map data is copyrighted to OpenStreetMap contributors under the Open Database License (https://www.openstreetmap.org/copyright/en). Base map style is copyrighted to Carto (www.carto.com) under a CC-BY 4.0 license (https://github.com/CartoDB/basemap-styles/blob/master/LICENSE.md).

To determine the potential of the smartphone data to distinguish between the three participant groups, we first quantified relative distances between individual GPS trajectories by using dynamic time warping (DTW) [50,51], while focusing on the trajectories during the walking phases (see section *GPS data analysis* for details). The resulting DTW dissimilarity matrix was submitted into a k-medoids clustering analysis to identify subgroups of participants who exhibited similar wayfinding styles, and it was tested how well the obtained clusters represent the three participant classes. This analysis showed that our sample is best described by three wayfinding clusters. Most members of the first cluster (n = 35; average distance to the cluster medoid = 0.08) were walking directly from POI to POI or showed only minor deviations from the most direct path to the POIs (e.g., detours). Members of the second cluster (n = 24; average distance to the cluster medoid = 0.09) typically took a less direct way to the POIs, including wrong turns at some intersections. The third cluster consisted of two members who took largely different paths relative to the rest of the sample (average distance to the cluster medoid = 0.17). A prototypical trajectory for each cluster can be found in Fig 2A–2C and the movement trajectories of the whole sample are provided in S1 Fig.

The correspondence between the obtained wayfinding clusters and the three participant classes was rather low (cluster purity = 0.475, Fig 2D). The majority of younger adults (n = 18) were members of the first cluster, which also included 10 healthy older adults and seven SCD patients. The second cluster mainly consisted of older adults (nine healthy older adults and 10 SCD patients), but also included five younger adults. The third cluster consisted of one healthy older adult and one SCD patient. Thus, interindividual differences in the movement trajectories were not sufficient to extract age- and health-related information about the sample within the context of our task, presumably due to a high context-dependency of the data (i.e., the unique characteristics of each track).

### Group separation improves considerably when being based on aggregated performance measures

**Fig 3:**
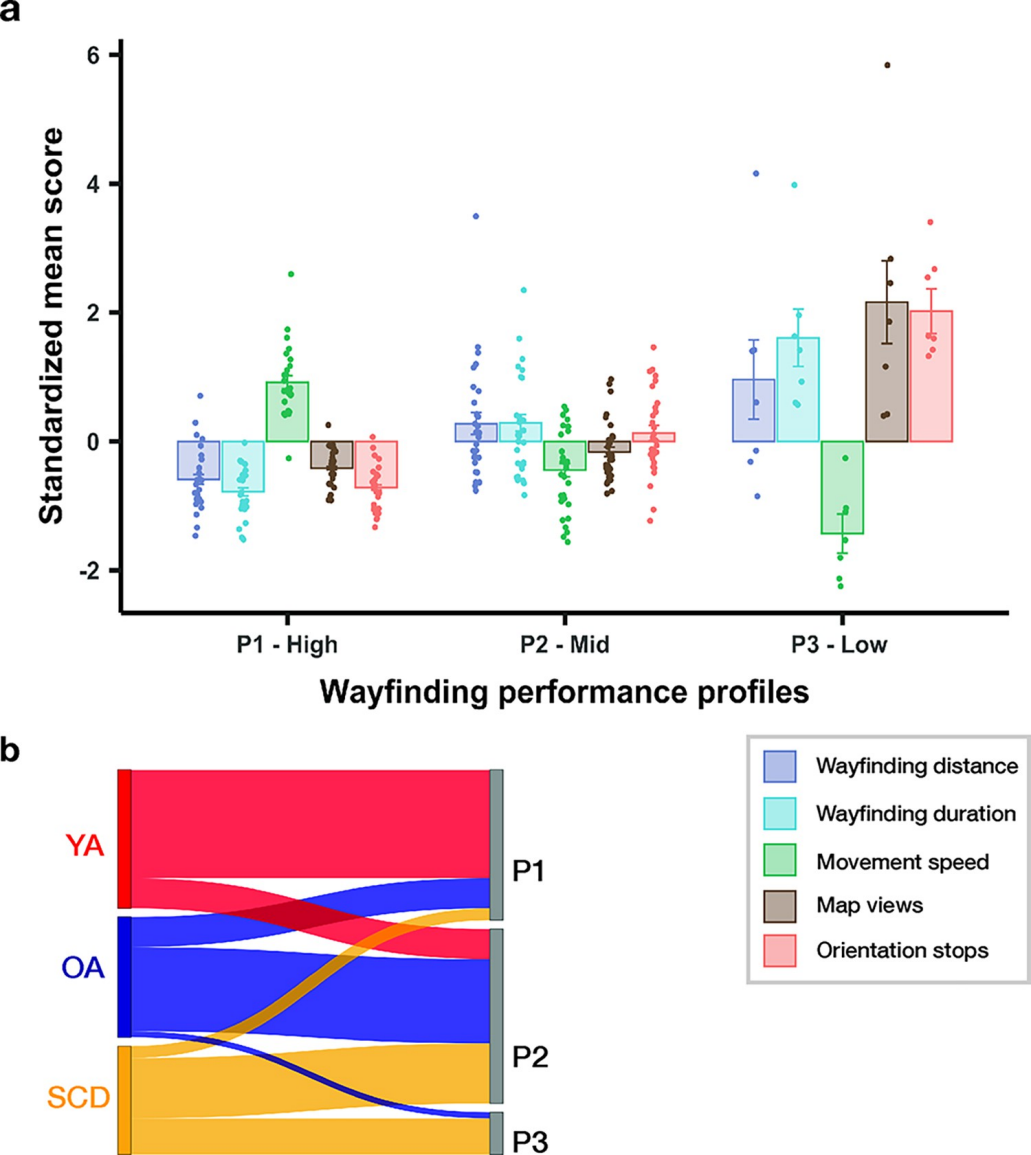
Three wayfinding performance profiles in our sample (P1: high-level; P2: mid-level; P3: low-level) as identified by a latent profile analysis (LPA) using five performance measures as input features. (a) Means (± standard error) of the z-scored performance measures for each profile (purple: wayfinding distance; light blue: wayfinding duration; green: movement speed; brown: number of map views during walking; orange: number of orientation stops). (b) Sankey diagram showing the distribution of our participant classes in each wayfinding performance profile. Red: younger adults; blue: healthy older adults; yellow: patients with subjective cognitive decline.

As a next step, we calculated five performance measures that were derived from the GPS data and user input data: 1) wayfinding distance; 2) wayfinding duration; 3) movement speed; 4) the number of help function calls during walking (map views); 5) the number of times the participants briefly stopped during walking (orientation stops) as a measure for increased cognitive processing demands when participants faced wayfinding difficulties [47,52]. A latent profile analysis (LPA) [53] was applied with these performance measures as input features to identify wayfinding performance profiles. We again tested how well the resulting profiles corresponded to the participant classes. This analysis showed that a model with three wayfinding performance profiles represented our sample best. The inspection of the single profiles with respect to their performance characteristics (Fig 3A) showed that participants expressing the first profile (n = 25) can be described as high-level performers who covered less distance, needed less time, and generally moved quicker during wayfinding. They also looked at the map less often during walking and had fewer orientation stops. The second profile was characterized by a mid-level performance on all measures (n = 29). Participants expressing the third profile (n = 7) covered more distance during walking, needed more time to complete the tracks, and moved slower. The biggest difference was evident for the number of times they called the help function and the number of the times they briefly stopped during walking, with both measures being considerably higher than in the other two profiles. Thus, participants with this profile can be described as low-level performers who had the biggest difficulties in finding the POIs.

Importantly, the participant classes were better represented by these wayfinding profiles than by the wayfinding clusters that we obtained from differences in the GPS trajectories (profile purity = 0.623, Fig 3B). The majority of the high-level performers were younger adults (n = 18). Only five healthy older adults and two SCD patients showed a similar high-performance pattern. Most of the healthy older adults (n = 14) and SCD patients (n = 10) were navigators who were characterized by a mid-level performance pattern in the context of our task. Five younger adults also fell in this category. A low-level performance was expressed by six SCD patients and one healthy older adult. This shows that different wayfinding performance parameters, which were extracted from GPS and user input data, are better suitable to identify age groups and SCD status than differences between movement trajectories.

### The number of orientation stops differs between healthy older adults and SCD patients

**Fig 4:**
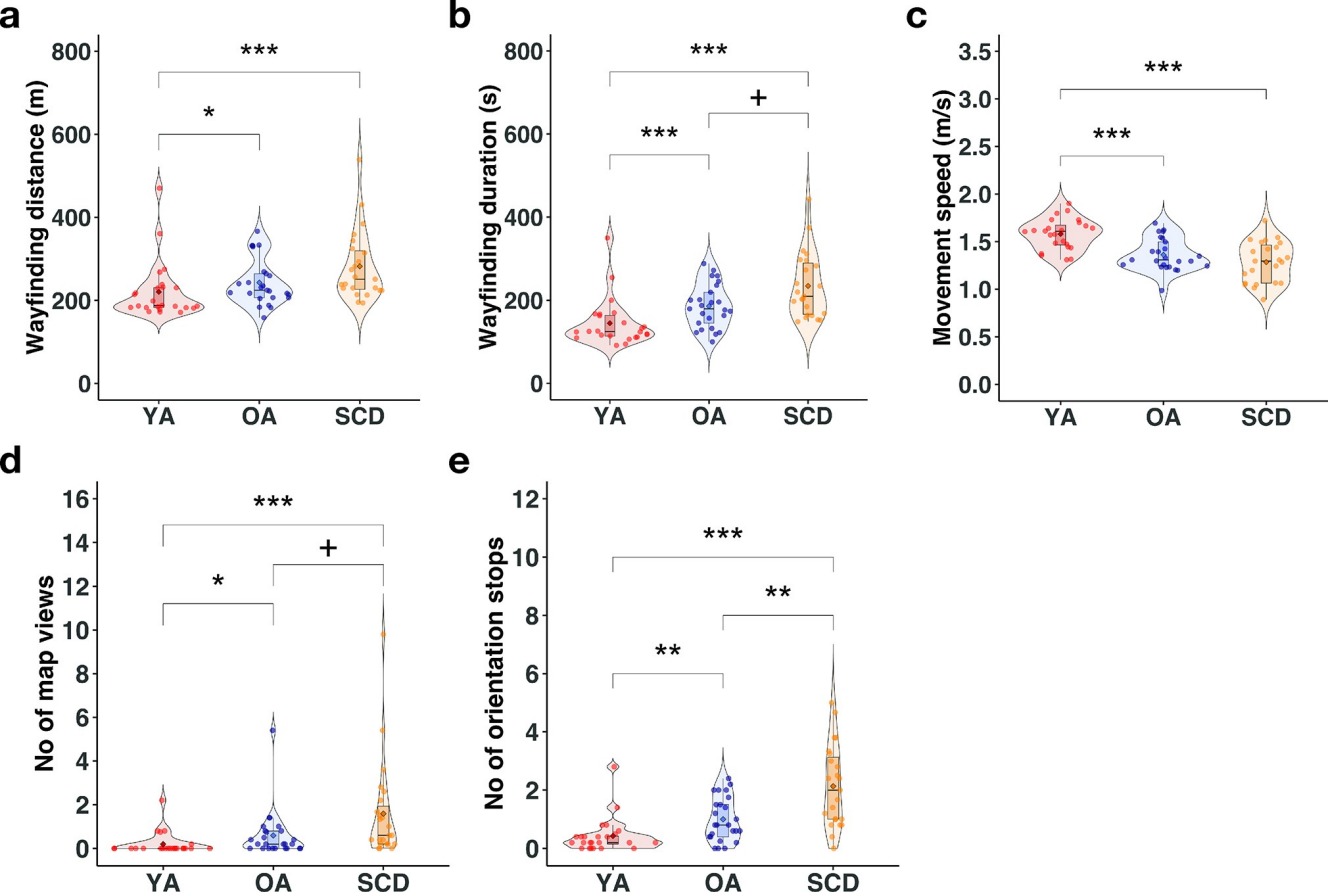
Results of the mixed effect model analyses. Performance of the three groups (red: younger adults; blue: healthy older adults; yellow: patients with subjective cognitive decline) for the (a) wayfinding distance; (b) wayfinding duration; (c) movement speed; (d) number of map views during walking; (e) number of orientation stops (divided by the number of completed tracks). The boxplot denotes the lower and upper quartile of the measure; center line the median; whiskers the 1.5x interquartile range; dots the individual data points; diamond shape the mean; ^+^ p < .10; * p < .05; ** p < .01; *** p < .001.

To investigate group differences on each performance measure in more detail, we fitted linear and generalized mixed effect (LME/GME) models with sex and campus familiarity as covariates and random intercepts for participant and track to the data (n = 72). This analysis showed that younger adults differed significantly from the two older participant groups on all performance measures (all p ≤ .018, Table 2). Compared with their older counterparts, they covered less distance, needed less time, had a higher movement speed, used the help function less often, and had fewer orientation stops during wayfinding (Fig 4). With respect to differences between healthy older adults and SCD patients, we found that SCD patients showed a significantly higher number of orientation stops than healthy older adults, β = 0.67, p = .007 (Fig 4E). They additionally tended to look at the map more often during walking, β = 0.82, p = .059 (Fig 4D), and tended to need more time to complete the task, β = 0.13, p = .088 (Fig 4B). This cannot be explained by walking more slowly since the average movement speed did not differ between the two older groups, β = -0.05, p = .371 (Fig 4C). SCD patients also did not cover more distance than the healthy older adults, β = 0.07, p = .208 (Fig 4A).

**Table 2:** Results of the mixed effect models estimating the fixed effects of group, campus familiarity, and sex on five performance measures.

	(a) Wayfinding distance			(b) Wayfinding duration			(c) Movement speed			(d) Number of map views			(e) Number of orientation stops		
**Fixed effects**															
	Est/Beta	95% CI	p	Est/Beta	95% CI	p	Est/Beta	95% CI	p	Est/Beta	95% CI	p	Est/Beta	95% CI	p
(Intercept)	5.368	5.007; 5.729	**< .001**	5.120	4.771; 5.468	**< .001**	1.310	1.196; 1.424	**< .001**	-0.655	-0.384; 0.758	.195	0.187	-0.384; 0.758	.521
Group YA	-0.127	-0.230; -0.024	**.018**	-0.283	-0.420; -0.146	**< .001**	0.223	0.123; 0.323	**< .001**	-1.243	-1.470; -0.369	**.016**	-0.919	-1.470; -0.369	**.001**
Group SCD	0.071	-0.038; 0.179	.208	0.128	-0.017; 0.273	.*088*	-0.048	-0.153; 0.056	.371	0.820	0.184; 1.157	.*059*	0.670	0.184; 1.157	**.006**
Familiarity	-0.003	-0.009; 0.002	.219	-0.008	-0.015; -0.001	**.038**	0.006	0.001; 0.011	**.031**	-0.048	-0.033; 0.019	**.050**	-0.007	-0.033; 0.019	.595
Sex Female	0.102	0.016; 0.188	**.023**	0.139	0.025; 0.253	**.020**	-0.054	-0.137; 0.029	.203	0.183	-0.105; 0.707	.618	0.301	-0.105; 0.707	.146
**Random effects**															
	Variance		SD	Variance		SD	Variance		SD	Variance		SD	Variance		SD
Participant	0.003		0.055	0.022		0.148	0.028		0.168	1.246		1.116	0.402		0.634
Track	0.154		0.393	0.130		0.361	0.002		0.130	0.003		0.063	0.000		0.000
**Model fit**															
Delta AIC	-9.25			-25.34			-23.32			-14.1			-25.94		
(Marginal / Pseudo) R^2^	0.033			0.108			0.277			0.265			0.268		

For all models, random intercepts were estimated per participant and track and confidence intervals were calculated using the Wald method. Model equations: performance measure ~ group + familiarity + sex + (1|participant) + (1|track). Performance measures were the (a) log-transformed wayfinding distance; (b) log-transformed wayfinding duration; (c) movement speed, (d) number of map views, and (e) number of orientation stops. P-values for fixed effects in the LME models (a-c) were calculated using the Satterthwaite’s approximation for degrees of freedom. For LME models (a-c) marginal R^2^ is reported. The GME models (d-e) were calculated using the maximum likelihood estimation and Pseudo R^2^ was calculated. For (d), a zero-inflated negative binomial distribution and for (e), a zero-inflated poisson distribution of the data was assessed and a log link function applied. Bold font indicates significant effects; italic font indicates statistical trends.

Taken together, we found that briefly stopping in order to orient themselves differed between healthy older adults and SCD patients. In contrast, general walking patterns, such as the overall distance that was covered and the movement speed, were largely similar among older adults, with the former presumably also being more influenced by the specific characteristics of the tracks. This is further supported by the variability between participants and tracks that we modeled as random effects in our models, showing that differences between tracks explained more variance than individual differences for wayfinding distance, whereas the reverse was true for the number of map views and orientation stops (Table 2, see also S2 Fig for the number of orientation stops on each track in healthy older adults and SCD patients and S3 Fig showing the location of every orientation stop for all three groups).

### The number of orientation stops predicts SCD status in older adults

**Fig 5:**
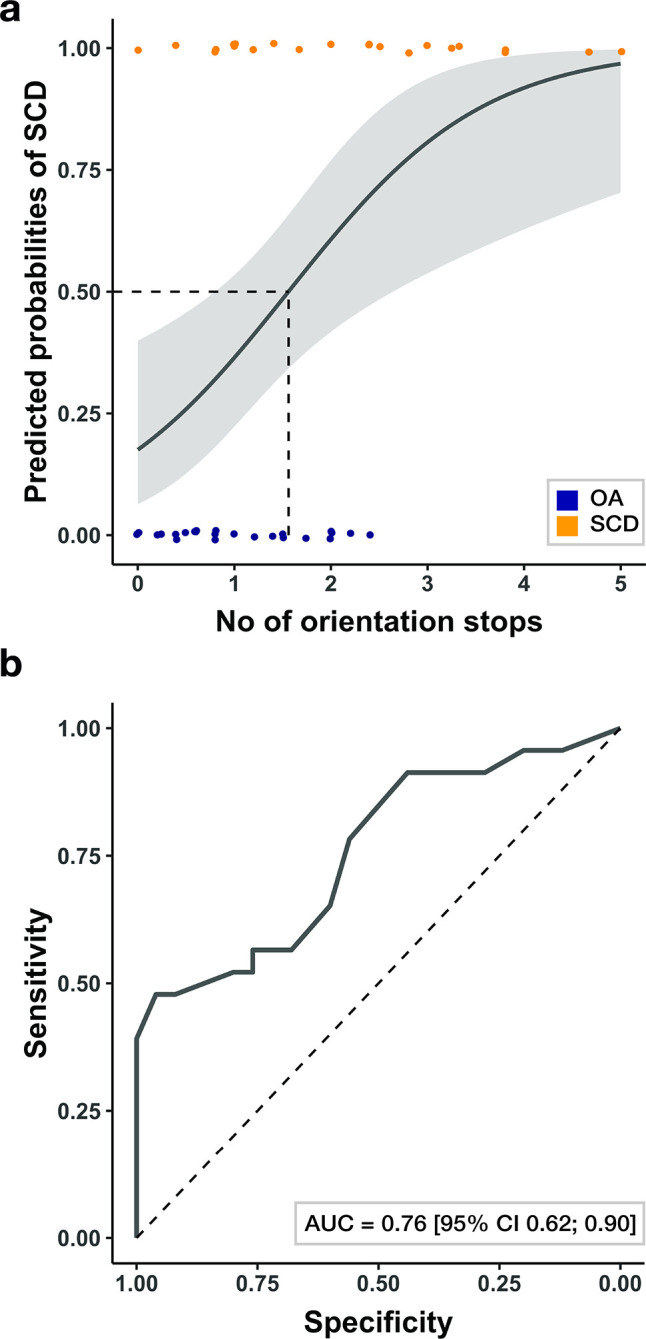
The number of orientation stops as predictor of SCD status in older adults. (a) Predicted probabilities of being classified as a SCD patient, as estimated in a logistic regression model with the number of orientation stops as input feature. Shaded areas denote the 95% confidence intervals of the predictions and dots the individual data points (blue: healthy older adults; yellow: patients with subjective cognitive decline). (b) Receiver Operating Characteristic (ROC) curve showing the diagnostic accuracy of the number of orientation stops for the detection of SCD in older adults.

As a next step, we were interested whether the average number of orientation stops across tracks that differed between healthy older adults and SCD patients, could be used to predict SCD status in older adults. The number of orientation stops was consequently fed into a logistic regression model with SCD status as outcome variable. The logistic regression model was significant, χ^2^ (1) = 8.1, p = .004 (see Fig 5A). A higher number of orientation stops was associated with significantly higher odds of being a patient with SCD than a healthy older adult, Odds Ratio = 2.70, 95% CI = 1.47–5.86, z = 2.85, p = .004. A leave-one-out (LOO) cross-validation confirmed that SCD status of unknown participants can be predicted above chance when using the predictions of the logistic regression model (accuracy = 0.67, CI = 0.53–0.81, chance level 0.50). Overall, 32 out of 48 individuals were correctly classified.

A receiver operating characteristic (ROC) analysis showed that the number of orientation stops differentiated healthy older adults and SCD patients with an area under curve (AUC) of 0.757 (SE = 0.07, 95% CI: 0.62–0.90, Fig 5B). A maximum sensitivity of 78% could be achieved with a specificity of at least 50%, and a maximum specificity of 80% could be achieved with a sensitivity of at least 50%. Thus, our results provide evidence that the number of orientation stops during wayfinding is predictive of SCD status in older adults.

### The number of orientation stops might be linked to executive functioning and environmental features in SCD patients

**Fig 6:**
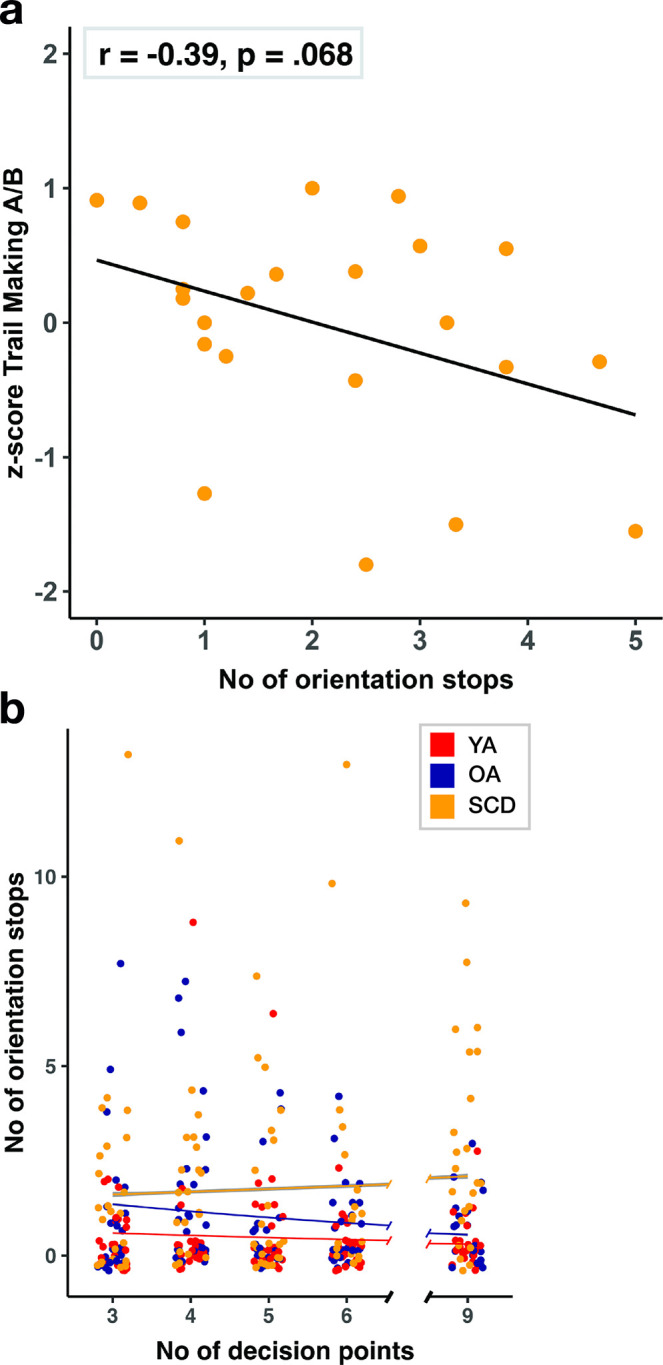
(A) Correlation between the average number of orientation stops and the z-scored Trail Making A/B ratio of the CERAD test battery in SCD patients. (B) Number of orientation stops relative to the number of decision points per track (red: younger adults; blue: healthy older adults; yellow: patients with subjective cognitive decline).

The individual number of orientation stops varied widely within SCD patients across tracks. To provide more insights into the cognitive processes that might be associated with this digital performance measure, we calculated in a post-hoc analysis the correlations between the number of orientation stops and the CERAD subtest scores that were available for this group (n = 23) [48]. This analysis showed that the number of orientation stops was moderately correlated with the MMSE score as a measure for general cognitive functioning (r = -.33), as well as with the Constructional Praxis Recall (r = -.32) and Savings test scores (r = -.31), which assess visual memory functioning. The highest correlation was observed for the Trail Making A/B score as a measure for executive functioning (task switching) [54], suggesting that a better performance in this test might be linked to fewer orientation stops during wayfinding in the real world in SCD patients (r = -.39, Fig 6A). However, none of the correlations reached statistical significance and should therefore be interpreted with caution (all p ≥ .068, see S1 Table).

Based on previous research indicating that individuals with dementia are more likely to become disoriented in environments with greater intersectional density [55], we next explored whether the number of decision points per track (defined as the number of intersections along the optimal path to the next PoI) were differently associated with the number of orientation stops between the groups. A GME model was fitted to analyze the number of orientation stops, incorporating fixed effects for the number of decision points and an interaction term for group and number of decision points (see Fig 6B). We found a significant main effect of decision points, β = -0.142, p = .015, which was further qualified by a significant interaction term in the SCD patient group, β = -0.157, p = .020. SCD patients stopped more often on tracks with a higher number of decision points compared with healthy older adults (see S2 Table for the full model statistics).

## Discussion

In this study, we evaluated the potential of smartphone data, recorded during wayfinding in the real world, to distinguish between healthy older adults and SCD patients who are known to possess a higher risk for developing dementia. We show that behavioral wayfinding performance indicators, derived from GPS and user input data, contain information about the participant’s age group and SCD status. We found that healthy younger adults showed an overall better performance in the wayfinding task compared to both older groups indicating that our real-world task is sensitive to age-related changes [56,57], whereas the differences between healthy older adults and SCD patients were more nuanced. Specifically, the number of brief stops while navigating to different locations in the environment differed between healthy older adults and SCD patients. This effect was strong enough to predict SCD status in older participants, rendering this performance measure as a promising digital footprint for dementia-related cognitive decline in real world settings. Ghosh and colleagues [44] found differences between AD dementia patients and older controls when passively tracking their movement trajectories over a period of two weeks in their own communities. In contrast, we show that SCD patients can be distinguished from cognitively healthy older adults during a mobile wayfinding task that was completed in less than half an hour. This group discrimination was driven by the amount of orientation stops during the wayfinding task. The absence of differences in movement trajectories between SCD and controls in our study compared to Gosh and colleagues [44] could be attributable to differences concerning the familiarity with the environment or other group characteristics as well as certain task specifics (experimental task vs naturalistic study). In our study, the number of orientation stops identified SCD patients with an AUC of 0.76, which is comparable to assessments of navigational performance in virtual environments [30,41]. However, in contrast to these earlier studies, we identified SCD patients on the basis of digital data obtained during a short and remotely performed task, which poses a major advantage for a potential future application in primary healthcare settings.

In contrast to previous VR navigation studies, we did not observe differences between healthy older adults and older adults at-risk for dementia with respect to the distances that were covered during wayfinding [23,24,41]. This might be related to the characteristics of the real-world environment in our study in comparison to the virtual environments used in previous studies, which often entailed relatively unconstrained spaces (e.g., virtual arenas) where participants have to rely predominantly on self-motion cues in order to estimate their position in the environment. This process (path integration) has been linked to computations of grid cells in the entorhinal cortex, which are affected by AD pathology at an early stage [23,24,58]. This shows laboratory-based studies using VR, focusing on pointing errors or wayfinding distances, might not yield the same results as studies in real-world settings. Cognitive changes might manifest differently in the real world where participants can additionally draw on various visual cues (e.g., landmarks) to aid their performance [59] and where their movement paths are naturally constrained by streets and barriers such as vegetation and objects (e.g., cars or fences), which might mask potential deficits in path integration.

A comparison between navigation performance in an environment in the real world and a virtual version of this environment would be required to investigate the transferability from lab-based navigation parameters to real-world outcome measures. This would allow understanding in more detail the (potentially complementary) sensitivity that can be achieved by these two approaches. One may hypothesize, for example, whether the number of orientation stops is correlated with VR-based measures or whether healthy older adults and SCD patients show similar wayfinding distances in a virtual version of an environment, given that previous research showed that performance in VR-based navigation tasks is related to real-world navigation performance [60]. Moreover, determining the performance of SCD patients on established measures of spatial navigation performance (e.g., virtual path integration or pointing tasks) would benefit the understanding of how individuals with SCD navigate. First indications on the nature of the navigation deficits in SCD patients have been provided by Chen and colleagues [30] showing that SCD patients, irrespective of navigational strategy (egocentric, allocentric, and mixed conditions), exhibit larger distances errors in a virtual morris water maze task. However, Hort and colleagues [61] did not observe differences in wayfinding distance between SCD patients and older adults in a navigation task using a circular arena.

In our study, group differences emerged in the number of orientation stops during wayfinding, in line with data from Taillade and colleagues [47] who observed a higher number of brief stops in healthy older adults compared with younger adults when they were asked to reproduce a path in the real world that was previously learned in VR. The authors concluded that more orientation stops during navigation might indicate impairments in executive functioning. Intact executive functioning is an important component of efficient navigation, for example, when switching between different navigation strategies or planning a route [27]. In addition to planning a route to reach a certain destination, monitoring the progress when walking towards the destination as well as decision making processes at intersections are all processes that strongly depend on executive functioning [62,63]. Moreover, due to age-related declines in sensorimotor processing that are partly compensated by cognitive strategies in order to reduce the risk of falling during walking, fewer cognitive resources are available for other operations with advancing age in such situations [64,65]. Thus, an increase in dual-task demands in a situation where the demands on cognitive processing are increased, for example, when translating information gathered from a map into concrete actions in an area that is not visited on a regular basis, might be one explanation for the higher number of orientation stops in the older age groups. This is in line with previous findings showing a detrimental effect of unsupported, active walking while solving a spatial navigation task in older compared with younger adults [66].

Recently, however, it has been shown that cognitive performance also seems to be maintained or even improved in some older adults in a dual-task walking condition [67]. This suggests that the ability to reallocate neural resources in dual-task situations might be a characteristic of successful cognitive aging. In line with this, Åhman and colleagues [68] observed worse performance in older adults with SCD compared with healthy older adults when they performed a dual-task that involved naming animals or months of the year backwards while walking. Similarly, Montero-Odasso and colleagues [69] demonstrated that a lower gait velocity while performing a cognitive task is predictive of conversion to dementia in patients with MCI. These findings suggest the observed wayfinding deficits in our study might be linked to declines in executive function-related components of spatial navigation and impairments in the processing of dual-task demands. Our correlational analysis between the number of orientation stops and the different test scores in the CERAD in SCD patients indicates that a higher number of orientation stops might be associated with declines in executive functioning, consequently impacting navigation behavior. We further found that the number of orientation stops was positively related to the number of decision points per track in SCD patients but not in the other groups. One plausible interpretation of this finding is that decision making processes at the intersections imposed increased cognitive demands, prompting compensatory stops in the SCD group. In line with this, Puthusseryppady and colleagues [55] found that dementia patients are more likely to lose orientation in environments with higher intersectional density and complexity. The precise contribution of different cognitive processes on digital assessments of wayfinding performance will be an important topic for future research.

The LPA in which five performance measures were used as input features showed that our sample was described best by three different wayfinding profiles. In terms of the overlap with our participant classes, we found that each wayfinding profile was expressed predominantly by one of the three groups, for example, most high-performing navigators were younger adults, whereas most of the low-performing navigators were SCD patients. However, in line with many previous studies on age-related cognitive decline, we also observed a certain amount of within-group variability in the SCD group, with quite a few SCD patients showing a mid-level performance pattern, and some even being classified as high-level performers [57,70–72]. Although the likelihood of progression to dementia is increased for older adults with SCD and even stronger for SCD patients in memory clinics [33], additional indicators need to be considered in order to predict who will eventually progress to dementia or not [73]. Individuals with SCD constitute a heterogeneous group due to diverse etiologies of the perceived cognitive decline, which may affect wayfinding patterns differently. Causes for SCD include progression to dementia, subthreshold psychiatric symptoms, as well as dysexecutive symptoms and some individuals reporting SCD will not be affected by a neurodegenerative disease at all [74–76].

To better explain within-group heterogeneity in wayfinding performance, future studies should assess APOE e4 status, as one of the strongest predictors for the future development of AD dementia. Previous research showed that young APOE e4 carriers show higher wayfinding distances in virtual spatial navigation tasks (e.g., Couglan and colleagues [41]) and APOE e4 status has also been linked to an increased subjective perception of cognitive decline [77]. Thus, one might hypothesize that SCD patients might differ from healthy older adults on additional wayfinding performance measures, depending on their genetic risk for AD. Information on biomarker status (tau and amyloid beta protein accumulation) and white matter hyperintensities will further help to improve the characterization of dementia and AD risk in older adults during navigation in the real world [78–80]. Moreover, longitudinal data are needed to determine which of the SCD patients are progressing to the MCI stage. Future studies should additionally assess lifestyle factors such as physical fitness [81] and cognitive [82] as well as vascular reserve factors [83,84] that contribute to preserved MTL function in old age, even in the face of emerging pathology.

Taken together, we show that digital markers, extracted from smartphone data acquired during a remotely performed wayfinding task that took less than half an hour to complete, are suggestive of the cognitive health status in older adults. The results of our study are a starting point for determining how smartphone data, acquired during wayfinding in the real world, can be used for the assessment of cognitive impairment in the context of dementia. We circumvented common limitations of VR technology, such as the increased susceptibility to cybersickness in older adults [40], and identified features from the data that differed between the groups and were less dependent on the characteristics of the environment (e.g., the specific track that was traveled), providing first indications how our task could be applied in different environments. For example, in a next version of the app, landmarks that lie in the vicinity of the user could be automatically selected as POIs in the wayfinding task, based on certain criteria such as the complexity of the tracks or number of decision points along the tracks. In addition, the potential of additional features, for example, from sensor data that track fine-motor movements [85], should be determined for a better classification of participant groups who are at risk for dementia. Multimodal data from smartphones and wearables have the potential to better account for different developmental trajectories and distinct subtypes that characterize the disease [26,86], especially in early, heterogenous stages like SCD.

With the rising adoption of smartphones and wearables in older age groups [8], data from mobile applications like the “Explore” app could ultimately be used as a screening tool to stratify subjects with regard to the need of extended cognitive and clinical diagnostics. Using digital performance measures might support the identification of individuals who may benefit from pharmacological treatments [6] or behavioral interventions [87,88]. Based on evidence showing that spatial navigation training enhances cognition and maintains MTL function in older adults [89–91], mobile tools like the “Explore” app might further provide the means to combine the positive effects of lifestyle interventions, for example, physical exercise [81] and cognitive training [89–91] to slow down the progression of dementia-related cognitive decline.

## Material and Methods

### Sample

In total, 72 participants took part in the study (24 younger adults, 25 cognitively healthy older adults, and 23 patients with SCD; see Table 1 for the characteristics of the sample). Patients with SCD were recruited from the DZNE memory clinic where they were referred to from their GPs or specialists and underwent a thorough neuropsychological assessment. The definition of SCD followed the criteria as proposed by Jessen and colleagues [31]: 1) self-report of lowered cognitive capacity in contrast to a formerly normal capacity that is not caused by a severe event; 2) unimpaired performance within the age-, sex-, and education-matched norms on neuropsychological tests used to assess MCI or prodromal AD; 3) exclusion of MCI, prodromal AD, or any other type of dementia; 4) the self-perceived decline of cognition cannot be attributed to a psychiatric, neurological, or medical disease apart from AD nor to medication or substance abuse. Recently, Jessen and colleagues [73] proposed six additional criteria of SCD that indicate a higher risk for a progression to dementia. In the present study, two of these criteria were fulfilled, namely seeking professional help and persistence of SCD over time (as indicated by reporting the perceived cognitive decline on at least two distinct occasions, for example, first to the GP and then again at the memory clinic after an average waiting time of six months).

Their cognitive health status was assessed by the consortium to establish a registry for Alzheimer’s disease test battery (CERAD) [49], including the Mini-Mental State Examination (MMSE) [92]. The remaining participants were recruited from the DZNE participant database. To be included as a cognitively healthy older adult, a score higher than 23 had to be obtained in the Montreal Cognitive Assessment (MoCA) [93,94]. All participants had normal or corrected-to-normal vision and none of them reported a history of psychiatric, neurological, or motoric diseases or use of medication that might affect task performance. All participants were community-dwelling individuals with no major mobility impairments as determined by the German version of the Life-Space-Assessment (LSA) [95,96]. This questionnaire measures the frequency of activities in five different life-spaces within the past month, ranging from the participant’s home to places outside of town, and further considers the level of independence with which these activities were completed. In addition, all participants were smartphone owners and, thus, can be considered as being experienced in using mobile devices. The study was approved by the ethics committee of the Otto von Guericke University of Magdeburg. Participants provided written informed consent to take part in the study and were paid for their participation.

### Assessment of the prior knowledge of the environment

Familiarity with the campus area around the DZNE Magdeburg was assessed prior to testing by using a self-developed questionnaire. In the first part of the questionnaire, participants were asked to report the number and frequency of previous visits on the campus, the number of buildings that were usually visited, as well as their self-rated familiarity with the campus area on a 7-point Likert scale. The questionnaire further contained three short tests assessing the spatial knowledge about the campus area, similar to previous approaches to measure spatial familiarity about a certain environment (S4 Fig) [97,98]. In the first test, participants had to indicate from a list of pictures showing 12 campus buildings (including the five POIs of the mobile wayfinding task), which of the buildings they recognize (maximum score: 12). In the second test, four picture triplets of the 12 buildings were presented. Here, participants had to choose for each triplet, which of the two lower buildings lies closer to the upper reference building (maximum score: 4). They also had the option to indicate that they don’t know the answer. In the third test, they saw a map of the campus with red dots at different locations and had to assign the 12 buildings to the corresponding dots, in this way identifying their location (maximum score: 12). The items from the three spatial knowledge tests showed an acceptable internal consistency (Cronbach’s α = 0.75) and the sum of correct answers in each test (total familiarity score) correlated highly with the self-reported familiarity (r = .70). The total familiarity score was subsequently included as covariate in the analyses, whenever applicable. Overall, our sample showed an intermediate familiarity with the environment and, importantly, there were no significant differences in the total familiarity score between the three groups, χ2(2) = 4.38, p = .112 (Table 1).

### Mobile wayfinding task

The mobile wayfinding task was implemented in the smartphone application “Explore”. For testing, the app was installed on two identical, DZNE-owned phones (Samsung A51) to ensure that all participants performed the task under the same conditions (e.g., in terms of display size or performance of the phone) and were not distracted by incoming calls or messages. The app was certified with the “ePrivacyApp” seal by ePrivacy (https://www.eprivacy.eu/en/privacy-seals/eprivacyapp/) to meet standard European data protection and security requirements.

In the mobile wayfinding task, five distinct buildings (points-of-interest, POI) had to be found on the campus area around the DZNE, with the DZNE serving as start and end point. This resulted in six different walking tracks that could be completed along a route that covered approximately 820 m. At the beginning of each track, a map was displayed that showed the location of the target POI (start phase). In addition, the participant’s location was displayed dynamically as a blue dot with a small arrow, indicating the pointing direction of the phone. For all tracks, the DZNE was marked on the map for reference. The visible section of the map was freely movable by the participant via dragging and the map could be zoomed via pinching. In the upper left corner of the display, a picture of the target POI was shown that could be closed and reopened. The start phase ended automatically, when the participant walked more than 8 m or after a time-out of 18 s without interaction on screen. The map could also be closed via pressing an “ok” button. After closing the map, the walking phase was initiated, where only footsteps were shown on screen together with two response buttons: a “I arrived” button, which initiated the capture phase when pressed, and a “I don´t know where I am” button, which initiated the help phase. When arriving at the POI, the participant had to press the “I arrived” button, which opened the phone camera, to scan a QR-Code that was placed at the entrance door of the building (capture phase). The “I don’t know where I am” button could be pressed when the participant felt lost and wanted to see the map again (help phase). After successfully scanning the QR code, the same procedure was initiated for the next POI. GPS data (latitude, longitude, and timestamp) were recorded by the app every two seconds, together with certain meta-data, such as the number of map views during walking. During the start and help phases as well as during the capture phases, sensor data (gyroscope and accelerometer) were recorded in addition, which are not considered here. The data were saved encrypted on the phone and were decrypted after downloading.

Before testing, participants performed a short practice track where they had to walk from a nearby parking lot to the DZNE. For this track, they were guided by the experimenter who introduced the task procedure and practiced all app features with them. Afterwards, participants were instructed to find the five POIs independently, while using the help function of the app only when absolutely necessary. After returning to the DZNE, participants were debriefed and asked if they used certain strategies during task performance.

### Data preprocessing

All data were analyzed using R v4.1.1. [99]. The GPS data were first cleaned by removing duplicated entries and checked for data artifacts, for example, when the GPS signal was lost. Data for nine tracks from eight participants (1 younger adult, 5 healthy older adults, 2 SCD patients) were removed due to technical problems or difficulties in following task instructions. For example, one participant reported in the debriefing, that she asked other pedestrians for directions on some tracks and the data for these tracks showed that she stopped at popular meeting spots. Hence, these tracks were excluded from the analyses. Three SCD patients discontinued participation prior to completing the round for various reasons (e.g., weather change or tiredness), resulting in incomplete data with five missing tracks for them. We further removed data that were recorded 15 m around the location of the POIs, when the QR code sign could already be seen. Data from the last track, where participants returned to the DZNE from the last POI, were not considered in the analyses because the DZNE also served as the starting point of the task. The five tracks in our analysis varied in length and number of decision points (intersections at which the participants could choose between different directions). The minimum distance that had to be covered for track 1 was approximately 147 m with five decision points (when following the most direct path). Track 2 covered 67 m with three decision points. Track 3 was the longest with 258 m minimum path length and nine decision points. Track 4 covered 149 m with four decision points and track 5 202 m with six decision points. Overall, 346 tracks were included in the analyses that considered data from the whole sample. For some of the analyses (GPS data analysis, LPA), only data from those participants were considered who completed all five tracks of the task (23 younger adults; 20 healthy older adults; 18 SCD patients; see S3 Table for the characteristics of the sub-sample), resulting in 305 tracks that were analyzed. The sub-sample showed similar characteristics as the whole sample in terms of age, sex distribution, and our questionnaire measures (e.g., the groups did not differ with respect to campus familiarity and general mobility, all p ≥ .272).

### GPS data analysis

To quantify differences in the movement trajectories between participants, we applied dynamic time warping (DTW) to the GPS data that were recorded during the walking phases, using the dtw package [100]. We only considered data from those participants who completed all tracks of the task because otherwise participants who completed the same subset of tracks would be considered more similar relative to those who completed all tracks. DTW is better suitable for time-series that vary in length compared with other similarity measures (e.g. Fréchet or Euclidean distance) and proved to be robust to outliers [50,51,101]. Additionally, DTW can reliably detect inter-individual differences within a group of trajectories [101]. The resulting DTW dissimilarity matrix (across all tracks) was normalized using a Min-Max normalization (range: 0–1; and then served as input feature in a k-medoid clustering analysis, as implemented in the *cluster* package [102]. We varied the number of possible clusters from three to seven and computed the average Silhouette coefficient as a measure for the distance between the resulting clusters (possible range: -1 to 1 with negative values indicating wrong cluster assignments and values near zero overlapping clusters [103]). We found three clusters to be the best choice for our sample (Silhouette coefficients per tested cluster number: 3 = 0.48; 4 = 0.28; 5 = 0.27; 6 = 0.31; 7 = 0.32). Next, the correspondence between the wayfinding clusters and participant classes was determined by calculating the cluster purity (possible range: 0–1 with higher values indicating lower within-cluster variation in terms of class labels).

### Performance measures analysis

For each track and participant, we extracted five performance measures from the GPS and user input data. First, the wayfinding distance was calculated by summarizing the haversine distance of adjacent coordinates. Second, the wayfinding duration was calculated as the sum of temporal differences between adjacent coordinates. Third, the movement speed was calculated by dividing the wayfinding distance by the wayfinding duration. Fourth, the number of help function calls during walking (map views) was extracted from the meta-data. Lastly, we calculated the number of orientation stops, defined as the number of times when the participants moved less than one meter in five seconds during walking, using the stay point algorithm [104]. The threshold of five seconds corresponded to the threshold that was used in previous research [47]. The threshold of one meter was used to account for noise in the GPS data.

To identify subgroups of navigators in our sample based on these performance measures, we performed a latent profile analysis (LPA) with the z-transformed scores of each measure, averaged across tracks, as input parameters [53]. Again, we only included data from those participants who completed all tracks of the task to allow a better comparability to the results of the GPS data analysis and because we used the mean scores across tracks for the analysis. LPA is also a data-driven approach that aims at the extraction of homogenous sub-samples and the resulting wayfinding profiles can be examined with respect to their performance characteristics. We fitted models with three to seven profiles to the data and evaluated them by comparing the Akaike information criterion (AIC), the Bayesian information criterion (BIC), the sample-adjusted BIC (SABIC), the bootstrap likelihood ratio test (BLRT), entropy values, and the number of observations in each profile (Table 3) [53,105]. The AIC was lowest for the model with seven profiles, whereas the BIC was lowest for the three-profile model. The lowest SABIC value was found for the seven-profile model. Entropy, which can vary between zero and one with higher values corresponding to better classification, was above 0.9 for models with four and more profiles. The bootstrap likelihood ratio test was not significant for the models with four, five, and six profiles. Hence, those models were excluded from further consideration. For the seven-profile model, more than three profiles contained 6 or fewer members, which were not deemed reliable [105]. Thus, the model with three wayfinding performance profiles was accepted. Again, we calculated the cluster purity to determine how well these profiles represented our participant classes.

**Table 3:** Model fit statistics of the latent profile analysis (LPA).

Model	AIC	BIC	SABIC	Entropy	BLRT p-value	N assigned to each profile
3-Profile	493.6	561.2	460.5	0.886	0.010	P1 = 25, P2 = 29, P3 = 7
4-Profile	489.1	569.3	449.8	0.912	0.089	P1 = 26, P2 = 24, P3 = 4, P4 = 7
5-Profile	485.0	577.9	439.5	0.924	0.139	P1 = 4, P2 = 16, P3 = 32, P4 = 2, P5 = 7
6-Profile	488.0	593.5	436.2	0.920	0.584	P1 = 15, P = 12, P3 = 19, P4 = 5, P5 = 7, P6 = 3
7-Profile	480.3	598.6	422.4	0.917	0.050	P1 = 13, P2 = 13, P3 = 19, P4 = 4, P5 = 3, P6 = 6, P7 = 3

AIC = Akaike information criterion; BIC = Bayesian information criterion; SABIC = sample-adjusted Bayesian information criterion; BLRT = bootstrap likelihood ratio test.

We then fitted, for each performance measure, mixed effect models, due to their capability to combine fixed and random effects, to the data, allowing us to assess group differences in more detail. For this analysis, data from the whole sample were considered since mixed effect models can handle missing data. For the log-transformed wayfinding distance and duration as well as the movement speed, we fitted a LME model to the data, using the *lme4* package [106]. For the number of map views during wayfinding and the number of orientation stops, GME models were fitted to the data, as implemented in the *glmmTMB* package [107] due to zero-inflation of the data. All models were estimated with sex and campus familiarity as covariates and track and participant as random intercepts (basic model). The fixed effect group (younger adults, healthy older adults, and patients with SCD) was then added to estimate the final model. The two models were compared by using the AIC with lower AIC values indicating better model fit [108,109]. The significance of all fixed effect predictors in the LME models was assessed using two-sided t-tests and the Satterthwaite’s approximation for degrees of freedom. The significance of the fixed effect predictors in the GME models was tested using two-sided z-tests. A significance threshold of α = .05 (uncorrected) was used.

Next, we used the average number of orientation stops across tracks, which was the performance measure on which SCD patients differed from healthy older adults, as predictor in a logistic regression model with SCD status as outcome variable in the subsample of older participants (n = 48). The prediction accuracy for data from unknown participants was assessed in a LOO cross-validation by fitting a logistic regression model in all but one participant and evaluating the prediction of the model parameter in the left-out (test) participant. This process was repeated 48 times so that each participant served as a test participant once. Afterwards, the diagnostic accuracy was assessed in a receiver operating characteristics (ROC) analysis with the *pROC* package [110]. The optimal cut-off values were determined by either maximizing sensitivity or specificity while constraining the other metric to a minimum of 0.5, using the *cutpointr* package [111].

We further calculated Pearson product-moment correlation coefficients between the number of orientation stops and the age-, sex-, and education-corrected z-scores from all available subtests of the CERAD test battery [49] in SCD patients (n = 23) to provide indications about which cognitive processes might be associated with this performance measure. As a last step, we fitted a GME model to the number of orientation stops with the fixed effects group and number of decision points as well as their interactions, sex and campus familiarity as covariates and participant as random intercepts. To evaluate the performance of this model, we compared it to a basic model without fixed effects using the AIC [108]. The significance of the fixed effect predictors was again assessed using two-sided z-tests. All p-values are reported uncorrected.

## Supporting information

S1 Fig Movement trajectories of the three participant groups (red: younger adults; blue: healthy older adults; yellow: patients with subjective cognitive decline) on (a) track 1, (b) track 2, (c) track 3, (d) track 4, and (e) track 5 of the mobile wayfinding task. Base map data is copyrighted to OpenStreetMap contributors under the Open Database License (https://www.openstreetmap.org/copyright/en). Base map style is copyrighted to Carto (www.carto.com) under a CC-BY 4.0 license (https://github.com/CartoDB/basemap-styles/blob/master/LICENSE.md).
(DOCX)

S2 Fig Number of orientation stops on each track in healthy older adults (blue) and patients with subjective cognitive decline (yellow). The boxplot denotes the lower and upper quartile of the measure; center line the median; whiskers the 1.5x interquartile range; dots the individual data points; diamond shape the mean.
(DOCX)

S3 Fig Location of all orientation stops (n = 390) across the entire route in healthy younger adults (red), healthy older adults (blue), and patients with subjective cognitive decline (yellow). Base map data is copyrighted to OpenStreetMap contributors under the Open Database License (https://www.openstreetmap.org/copyright/en). Base map style is copyrighted to Carto (www.carto.com) under a CC-BY 4.0 license (https://github.com/CartoDB/basemap-styles/blob/master/LICENSE.md).
(DOCX)

S4 Fig Spatial memory tests implemented in the familiarity questionnaire to assess the participants’ prior knowledge of the campus area (maximum score: 28). (a) Landmark recognition test: First, participants had to indicate from a list of pictures showing 12 campus buildings (including the 5 PoIs of the mobile wayfinding task), which of the buildings they recognize (shown are 4 example buildings). (b) Distance estimation test: Next, they saw 4 triplets of the 12 buildings and were asked to indicate, which of the two buildings in the lower row lies closer to the reference building in the upper row (shown is one example triplet). (c) Map test: Finally, for the buildings they knew, they had to assign the buildings from the landmark recognition test to dots on a map of the campus, in this way identifying their location. The campus map used in the map drawing test displayed in S4C Fig was created by Nadine Diersch (last author) for the purpose of the study.
(DOCX)

S1 Table Pearson product-moment correlation coefficients (df = 21, a df = 18) between the number of orientation stops and the age-, sex-, and education-corrected z-scores from all available subtests of the CERAD test battery in patients with subjective cognitive decline (SCD). (DOCX)

S2 Table Results of the generalized mixed effect (GME) model estimating the fixed effects of group, number of decision points, interaction between group and number of decision points, campus familiarity, and gender on the number of orientation stops. Random effects were estimated per participant. Model equations: number of orientation stops ~ group * decision points + familiarity + sex + (1|participant). The GME model was calculated using the maximum likelihood estimation and Pseudo R^2^ was calculated. A zero-inflated poisson distribution of the data was assessed and a log link function applied.
(DOCX)

S3 Table Sample characteristics (descriptives, mean scores ± SD) of the three participant groups (YA: younger adults; OA: healthy older adults; SCD: patients with subjective cognitive decline), when only considering those individuals who completed all five tracks in the mobile wayfinding task (N = 61). The CERAD composite score was calculated using the age-, sex-, and education-corrected z-scores from six different subtests (Boston Naming Test, verbal fluency, word list learning, word list recall, word list savings, and constructional praxis, see Chandler and colleagues [49]). The groups did not differ in the listed attributes. Age differences between healthy older adults and patients with SCD were tested using a two-sided Welch two-sample t-test, t (25.85) = 0.40, p = .694, d = 0.14. Sex differences were tested using a χ2 test, χ2 (2) = 0.45, p = .799, d = 0.17. Differences in campus familiarity were tested using a Kruskal-Wallis rank-sum test, χ2 (2) = 2.60, p = .272, η^2^ = 0.01; Life-space assessment score differences using an analysis of variance, F(2,58) = 0.33, p = .718, η^2^ = 0.01.
(DOCX)
