# Supplementary material for: Identifying older adults at risk for dementia based on smartphone data obtained during a wayfinding task in the real world
Source: PLOS Digit Health. 2024 Oct 3;3(10):e0000613. doi: 10.1371/journal.pdig.0000613 (PMC11449328; doi:10.1371/journal.pdig.0000613)
Supplement: S1 Fig — Movement trajectories of the three participant groups (red: younger adults; blue: healthy older adults; yellow: patients with subjective cognitive decline) on (a) track 1, (b) track 2, (c) track 3, (d) track 4, and (e) track 5 of the mobile wayfinding task. Base map data is copyrighted to OpenStreetMap contributors under the Open Database License (https://www.openstreetmap.org/copyright/en). Base map style is copyrighted to Carto (www.carto.com) under a CC-BY 4.0 license (https://github.com/CartoDB/basemap-styles/blob/master/LICENSE.md). (DOCX) [file pdig.0000613.s001.docx]

**
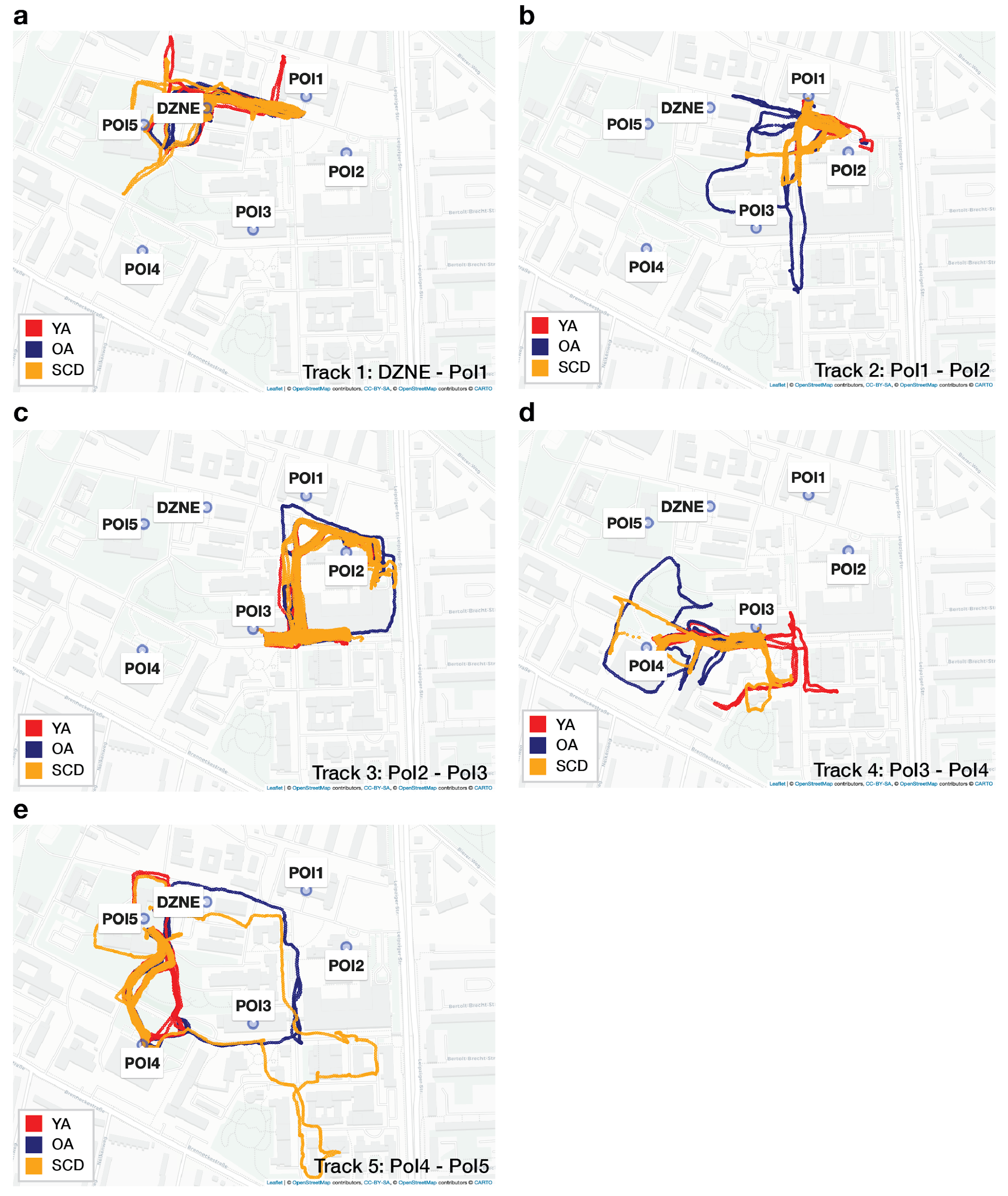
**

**S1 Fig.** Movement trajectories of the three participant groups (red: younger adults; blue: healthy older adults; yellow: patients with subjective cognitive decline) on (a) track 1, (b) track 2, (c) track 3, (d) track 4, and (e) track 5 of the mobile wayfinding task. Base map data is copyrighted to OpenStreetMap contributors under the Open Database License (<https://www.openstreetmap.org/copyright/en>). Base map style is copyrighted to Carto (www.carto.com) under a CC-BY 4.0 license (<https://github.com/CartoDB/basemap-styles/blob/master/LICENSE.md>).
