# Supplementary material for: Identifying older adults at risk for dementia based on smartphone data obtained during a wayfinding task in the real world
Source: PLOS Digit Health. 2024 Oct 3;3(10):e0000613. doi: 10.1371/journal.pdig.0000613 (PMC11449328; doi:10.1371/journal.pdig.0000613)
Supplement: S2 Fig — Number of orientation stops on each track in healthy older adults (blue) and patients with subjective cognitive decline (yellow). The boxplot denotes the lower and upper quartile of the measure; center line the median; whiskers the 1.5x interquartile range; dots the individual data points; diamond shape the mean. (DOCX) [file pdig.0000613.s002.docx]

**
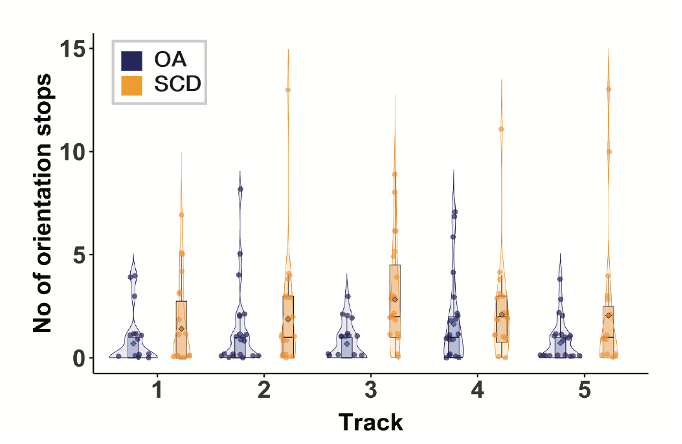
 S2 Fig.** Number of orientation stops on each track in healthy older adults (blue) and patients with subjective cognitive decline (yellow). The boxplot denotes the lower and upper quartile of the measure; center line the median; whiskers the 1.5x interquartile range; dots the individual data points; diamond shape the mean.
