# Supplementary material for: Identifying older adults at risk for dementia based on smartphone data obtained during a wayfinding task in the real world
Source: PLOS Digit Health. 2024 Oct 3;3(10):e0000613. doi: 10.1371/journal.pdig.0000613 (PMC11449328; doi:10.1371/journal.pdig.0000613)
Supplement: S3 Fig — Location of all orientation stops (n = 390) across the entire route in healthy younger adults (red), healthy older adults (blue), and patients with subjective cognitive decline (yellow). Base map data is copyrighted to OpenStreetMap contributors under the Open Database License (https://www.openstreetmap.org/copyright/en). Base map style is copyrighted to Carto (www.carto.com) under a CC-BY 4.0 license (https://github.com/CartoDB/basemap-styles/blob/master/LICENSE.md). (DOCX) [file pdig.0000613.s003.docx]

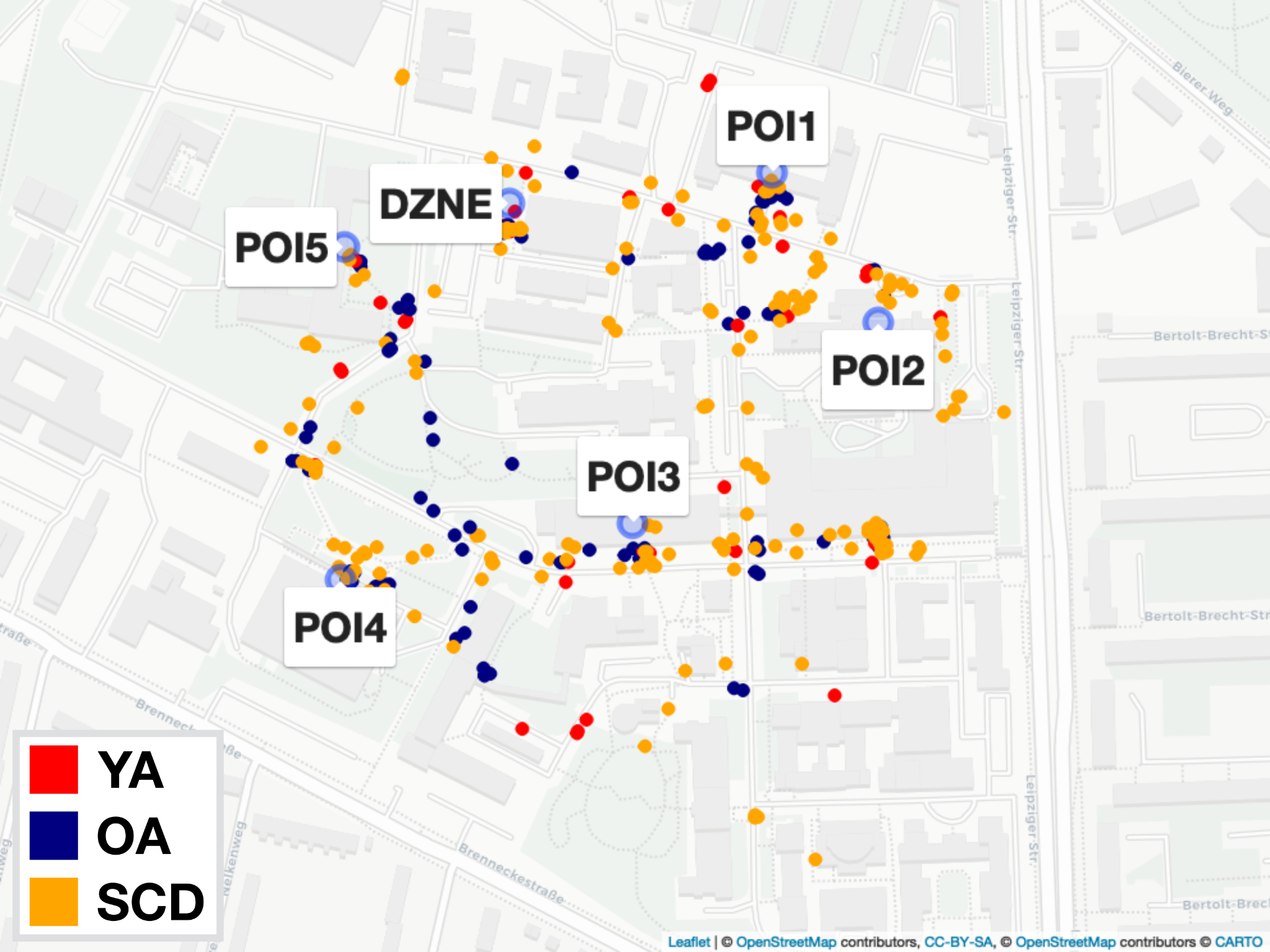
 **S3 Fig.** Location of all orientation stops (n=390) across the entire route in healthy younger adults (red), healthy older adults (blue), and patients with subjective cognitive decline (yellow). Base map data is copyrighted to OpenStreetMap contributors under the Open Database License (<https://www.openstreetmap.org/copyright/en>). Base map style is copyrighted to Carto (www.carto.com) under a CC-BY 4.0 license (<https://github.com/CartoDB/basemap-styles/blob/master/LICENSE.md>).
