# Supplementary material for: Identifying older adults at risk for dementia based on smartphone data obtained during a wayfinding task in the real world
Source: PLOS Digit Health. 2024 Oct 3;3(10):e0000613. doi: 10.1371/journal.pdig.0000613 (PMC11449328; doi:10.1371/journal.pdig.0000613)
Supplement: S4 Fig — (a) Landmark recognition test: First, participants had to indicate from a list of pictures showing 12 campus buildings (including the 5 PoIs of the mobile wayfinding task), which of the buildings they recognize (shown are 4 example buildings). (b) Distance estimation test: Next, they saw 4 triplets of the 12 buildings and were asked to indicate, which of the two buildings in the lower row lies closer to the reference building in the upper row (shown is one example triplet). (c) Map test: Finally, for the buildings they knew, they had to assign the buildings from the landmark recognition test to dots on a map of the campus, in this way identifying their location. The campus map used in the map drawing test displayed in S4C Fig was created by Nadine Diersch (last author) for the purpose of the study. (DOCX) [file pdig.0000613.s004.docx]

**
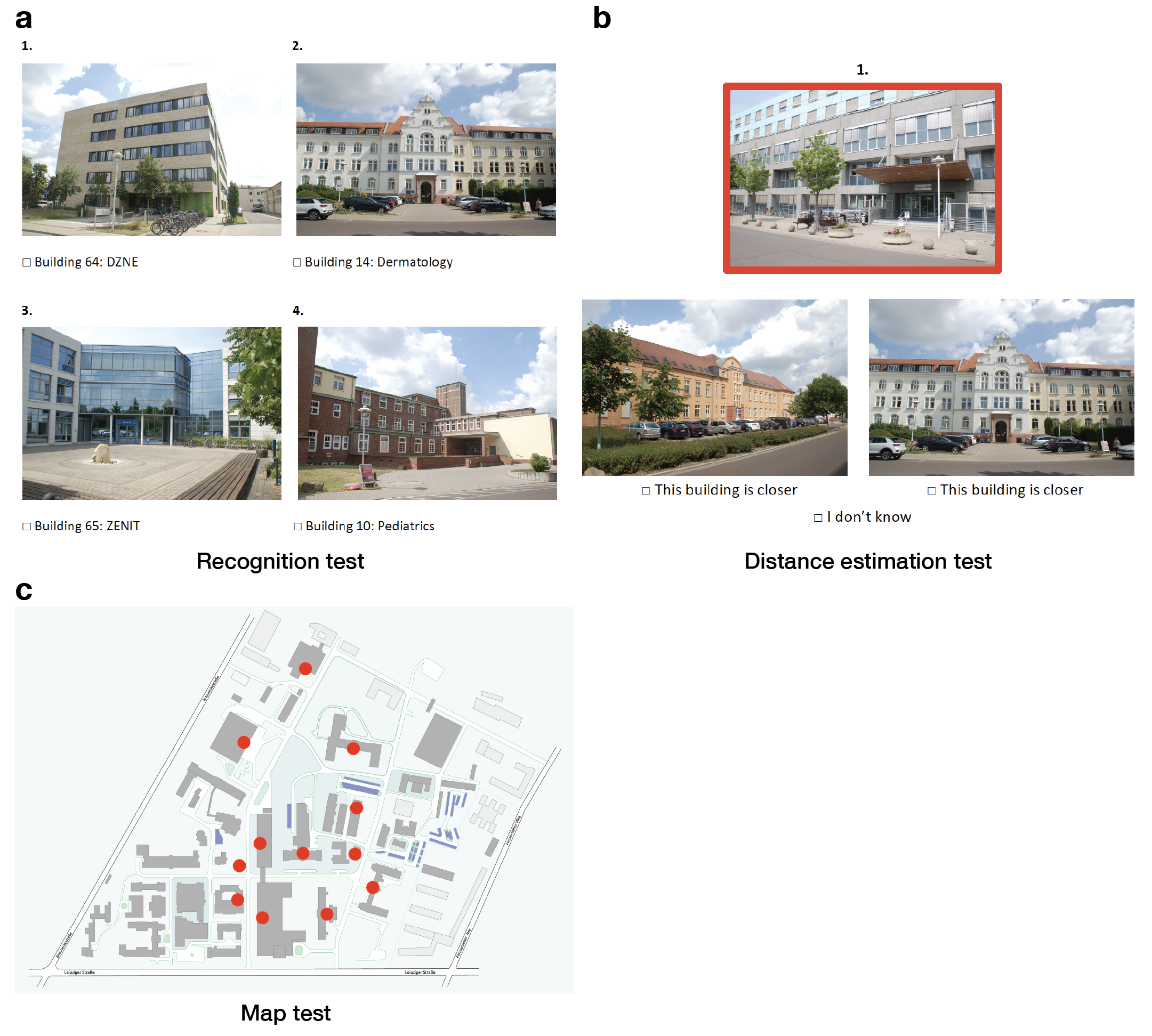
 S4 Fig.** Spatial memory tests implemented in the familiarity questionnaire to assess the participants’ prior knowledge of the campus area (maximum score: 28). (a) Landmark recognition test: First, participants had to indicate from a list of pictures showing 12 campus buildings (including the 5 PoIs of the mobile wayfinding task), which of the buildings they recognize (shown are 4 example buildings). (b) Distance estimation test: Next, they saw 4 triplets of the 12 buildings and were asked to indicate, which of the two buildings in the lower row lies closer to the reference building in the upper row (shown is one example triplet). (c) Map test: Finally, for the buildings they knew, they had to assign the buildings from the landmark recognition test to dots on a map of the campus, in this way identifying their location. The campus map used in the map drawing test displayed in S4C Fig was created by Nadine Diersch (last author) for the purpose of the study.
