# Supplementary material for: Identifying older adults at risk for dementia based on smartphone data obtained during a wayfinding task in the real world
Source: PLOS Digit Health. 2024 Oct 3;3(10):e0000613. doi: 10.1371/journal.pdig.0000613 (PMC11449328; doi:10.1371/journal.pdig.0000613)
Supplement: S1 Table — (DOCX) [file pdig.0000613.s005.docx]

| **CERAD Subtest** | **r** | **p-value** |
| --- | --- | --- |
| Verbal Fluency (animals) | -.10 | .640 |
| Boston Naming Test | .20 | .371 |
| Mini-Mental Status Examination | -.33 | .122 |
| Word List Learning | -.26 | .228 |
| Word List Recall | -.18 | .412 |
| Word List Savings | .03 | .901 |
| Word List Discrimination | .14 | .524 |
| Constructional Praxis Drawing | .21 | .332 |
| Constructional Praxis Recall | -.32 | .136 |
| Constructional Praxis Savings | -.31 | .144 |
| Verbal Fluency (words)^a^ | .16 | .494 |
| Trail Making Test A | -.07 | .756 |
| Trail Making Test B | -.29 | .184 |
| Trail Making Test A/B | -.39 | .068 |

**S1 Table.** Pearson product-moment correlation coefficients (df = 21, a df = 18) between the number of orientation stops and the age-, sex-, and education-corrected z-scores from all available subtests of the CERAD test battery in patients with subjective cognitive decline (SCD).
