# Supplementary material for: Identifying older adults at risk for dementia based on smartphone data obtained during a wayfinding task in the real world
Source: PLOS Digit Health. 2024 Oct 3;3(10):e0000613. doi: 10.1371/journal.pdig.0000613 (PMC11449328; doi:10.1371/journal.pdig.0000613)
Supplement: S2 Table — Random effects were estimated per participant. Model equations: number of orientation stops ~ group * decision points + familiarity + sex + (1|participant). The GME model was calculated using the maximum likelihood estimation and Pseudo R2 was calculated. A zero-inflated poisson distribution of the data was assessed and a log link function applied. (DOCX) [file pdig.0000613.s006.docx]

| **Fixed effects** | | | | | |
| --- | --- | --- | --- | --- | --- |
|  | Est/Beta | 95% CI | | p | |
| Intercept | 0.928 | 0.123; 1.733 | | **.024** | |
| Group YA | -1.203 | -2.302; -0.104 | | **.032** | |
| Group SCD | -0.137 | -0.973; 0.700 | | .749 | |
| Decision Points | -0.142 | -0.257; -0.027 | | **.015** | |
| Group YA x Decision Points | 0.058 | -0.132; 0.248 | | .550 | |
| Group SCD x Decision Points | 0.157 | 0.0247; 0.289 | | **.020** | |
| Familiarity | -0.008 | -0.034; 0.018 | | .543 | |
| Sex Female | 0.297 | 0.016; 0.188 | | .143 | |
| **Random effects** | | | | | |
|  | Variance | SD |  | | |
| Participant | 0.373 | 0.611 |  | | |
| **Model Fit** | | | | | |
| Delta AIC | -29.56 | Pseudo R^2^ | | | 0.285 |

**S2 Table.** Results of the generalized mixed effect (GME) model estimating the fixed effects of group, number of decision points, interaction between group and number of decision points, campus familiarity, and gender on the number of orientation stops. Random effects were estimated per participant. Model equations: number of orientation stops ~ group * decision points + familiarity + sex + (1|participant). The GME model was calculated using the maximum likelihood estimation and Pseudo R² was calculated. A zero-inflated poisson distribution of the data was assessed and a log link function applied.
