# Supplementary material for: Identifying older adults at risk for dementia based on smartphone data obtained during a wayfinding task in the real world
Source: PLOS Digit Health. 2024 Oct 3;3(10):e0000613. doi: 10.1371/journal.pdig.0000613 (PMC11449328; doi:10.1371/journal.pdig.0000613)
Supplement: S3 Table — The CERAD composite score was calculated using the age-, sex-, and education-corrected z-scores from six different subtests (Boston Naming Test, verbal fluency, word list learning, word list recall, word list savings, and constructional praxis, see Chandler and colleagues [49]). The groups did not differ in the listed attributes. Age differences between healthy older adults and patients with SCD were tested using a two-sided Welch two-sample t-test, t (25.85) = 0.40, p = .694, d = 0.14. Sex differences were tested using a χ2 test, χ2 (2) = 0.45, p = .799, d = 0.17. Differences in campus familiarity were tested using a Kruskal-Wallis rank-sum test, χ2 (2) = 2.60, p = .272, η2 = 0.01; Life-space assessment score differences using an analysis of variance, F(2,58) = 0.33, p = .718, η2 = 0.01. (DOCX) [file pdig.0000613.s007.docx]

|  | **YA** | **OA** | **SCD** |
| --- | --- | --- | --- |
| n | 23 | 20 | 18 |
| Age | 24.4 ± 2.29 | 66.0 ± 3.79 | 65.2 ± 6.87 |
| No of female | 12 | 9 | 10 |
| Campus familiarity  (max. score: 28) | 12.9 ± 9.30 | 13.4 ± 8.40 | 9.2 ± 6.59 |
| Life-Space Assessment (max. score: 120) | 81.3 ± 12.3 | 84.8 ± 16.1 | 83.4 ± 14.1 |
| Cognitive screening scores | -- | MoCA: 28.4 ± 1.09 | MMSE: 29.0 ± 1.19  CERAD: 0.24 ± 0.54 min = -0.75 |

**S3 Table.** Sample characteristics (descriptives, mean scores ± SD) of the three participant groups (YA: younger adults; OA: healthy older adults; SCD: patients with subjective cognitive decline), when only considering those individuals who completed all five tracks in the mobile wayfinding task (N = 61). The CERAD composite score was calculated using the age-, sex-, and education-corrected z-scores from six different subtests (Boston Naming Test, verbal fluency, word list learning, word list recall, word list savings, and constructional praxis, see Chandler and colleagues [1]). The groups did not differ in the listed attributes. Age differences between healthy older adults and patients with SCD were tested using a two-sided Welch two-sample t-test, t (25.85) = 0.40, p = .694, d = 0.14. Sex differences were tested using a χ2 test, χ2 (2) = 0.45, p = .799, d = 0.17. Differences in campus familiarity were tested using a Kruskal-Wallis rank-sum test, χ2 (2) = 2.60, p = .272, η^2^ = 0.01; Life-space assessment score differences using an analysis of variance, F(2,58) = 0.33, p = .718, η^2^ = 0.01.

**References**

1. Chandler MJ, Lacritz LH, Hynan LS, Barnard HD, Allen G, Deschner M, et al. A total score for the CERAD neuropsychological battery. Neurology. 2005 Jul 12;65(1):102-6
